# Cultural mismatch and accelerated epigenetic age during the transition to college

**DOI:** 10.1016/j.bbih.2025.100989

**Published:** 2025-04-07

**Authors:** Yolanda Vasquez-Salgado, Gabrielle Halim, Angel E. Morales, Katelan Galvan, Teresa Seeman, Steve Cole

**Affiliations:** a California State University, Northridge, United States; b University of California, Los Angeles, United States

**Keywords:** Home-school cultural value mismatch, Cultural mismatch, Family obligation, Epigenetic age, Epigenetic age acceleration, Biological aging, Historically marginalized students, Transition to college

## Abstract

Previous literature has highlighted *home-school cultural value mismatch* – a cultural mismatch between interdependent family obligations and independent academic obligations, as a psychosocial stressor among first-generation college students from historically marginalized backgrounds. However, no studies to date have examined its association with an objective biomarker of health. Given accumulating evidence linking higher psychosocial stress to accelerated *epigenetic age* – a measure of one's biological age based on DNA methylation levels, we hypothesized that higher levels of cultural mismatch would be associated with accelerated epigenetic age. In this Transition to College Study, historically marginalized students (*N* = 64; *M*_age_ = 18.0; *SD* = 0.4; 82.8 % Latinx; 87.5 % first-generation college) completed an online survey and provided a salivary sample during their first semester at a public four-year university. GrimAge, FitAge and DunedinPACE, second and third-generation epigenetic aging measures, were used for analysis. Hierarchical linear regressions, controlling for chronological age, ethnicity, biological sex, body-mass-index, smoking and alcohol use, and parental socioeconomic status, were used to test our hypothesis. Results indicated that higher levels of mismatch between family and academic obligations were associated with accelerated epigenetic age as measured by GrimAge and FitAge, but not DunedinPACE. Our findings highlight a novel association between this mismatch and biomarkers known to predict mortality and future disease risk. Implications for research and interventions in higher education are discussed.

## Highlights

• Mismatch between home and school cultures is a stressor among historically marginalized (HM) students.
• In a sample of 64 HM students, we assessed cultural mismatch and its relation to epigenetic clocks: GrimAge, FitAge, and DunedinPACE.
• GrimAge and FitAge were accelerated by 1.3 and 2.0 years, respectively, per every 1-SD increase in cultural mismatch.
• Cultural mismatch may contribute to future health risks by accelerating biological aging.

## 1 Introduction

First-generation college students often express a desire to assist and spend time with family yet feel constrained by the demands of their university commitments. This phenomenon has been documented in the literature as *home-school cultural value mismatch* – a cultural mismatch between interdependent family obligations and independent academic obligations. This mismatch can have a surprising impact on the overall quality of life and stress levels of first-generation college students from historically marginalized backgrounds:

I was … [pauses to clear throat] … a big help to my family when I was there … it stresses me out … I’m always like under that constant pressure … I could be … helping … but yet I’m here … it’s … hard. [Vasquez-Salgado et al., 2015, p. 292]

I remember … multiple times … half of me would want to go and … half of me would be so strict on myself to force myself to stay, but … I couldn’t do my work because in my mind I was like I should have gone home, I should have done this and that … [ Vasquez-Salgado et al., 2015, p. 291]

The quotes above represent Latinx first-generation college students' lived experiences with feeling torn between engaging in family obligations or academic obligations during their first year of university. Their words, “stress”, “constant pressure” and “in my mind … I should have done this and that” elucidate a sense of psychosocial stress. Qualitative and survey research suggests that this cultural mismatch is not only experienced by Latinx first-generation college students but also by first-generation college students from other backgrounds (Vasquez-Salgado et al., 2015, 2021) during the transition to college. Though this mismatch has been linked to an increase in students’ vulnerability to psychosocial stress (Valle and Covarrubias, 2024; Vasquez-Salgado et al., 2015, 2021), and experimentally induced behavioral processes connected to stress (Vasquez-Salgado et al., 2018), no work has unveiled its connection to objective markers of health linked to psychosocial stress. The current study intends to fill this gap by examining the association between home-school cultural value mismatch and biological aging.

### 1.1 Epigenetic age

Epigenetic age is a measure of one's biological age based on DNA methylation levels and is highly correlated with chronological age (number of years an individual has lived; Duan et al., 2022). However, epigenetic age can sometimes be “accelerated” relative to chronological age, and such epigenetic age acceleration has been linked to increased risk of future disease and mortality. A “zero value” of epigenetic age acceleration indicates alignment in biological and chronological age, negative values indicate slower biological aging, and positive values indicate accelerated epigenetic aging. The concept of epigenetic age acceleration implies that individuals with the same chronological age can vary in biological age, sparking a scientific quest to define the health significance of such effects, as well as their sociocultural determinants; the latter represents the goal of the current research.

Recent technological advances have enabled scientists to assess biological age through an array of “epigenetic clocks,” which are composite measures of genomic DNA methylation (DNAm) marks that correlate with aging-related phenotypes, such as chronological age, illness, and mortality (Li et al., 2022). First-generation epigenetic clocks used DNAm to predict chronological age (Horvath, 2013), whereas second-generation models used DNAm to predict both chronological age and health attributes (e.g., functional decline, time to death; Lu et al., 2019; McGreevy et al., 2023). The development of DunedinPACE marked the emergence of third-generation clocks, which were specifically designed to predict the pace of aging directly (Belsky et al., 2022). A DunedinPACE value of 1 indicates an average pace of aging with the population, whereas values lower or higher than 1 indicate slower or faster pace, respectively. This study employs second- and third-generation clocks - namely, GrimAge, FitAge, and DunedinPACE - due to their demonstrated ability to predict disease risk and mortality (Belsky et al., 2022; Lu et al., 2019; McCrory et al., 2021; McGreevy et al., 2023).

Accumulating evidence suggests psychosocial variables such as socioeconomic status (Markon et al., 2024; Potente et al., 2023), early life adversity (Belsky et al., 2022; Klopack et al., 2022) and trauma (Holloway et al., 2023; Palma-Gudiel et al., 2020) are associated with accelerated epigenetic aging in healthy community samples. However, no research has examined the association between cultural mismatch and epigenetic aging during the transition to college – a critical period of development in young, emerging adults (Arnett, 2000).

### 1.2 Theoretical perspective

Cultural mismatch theory asserts that first-generation college students (i.e., students whose parents have not received a bachelor's degree) are often raised with interdependent values (e.g., focus on family, group goals) that mismatch with the independent values of the university environment (e.g., focus on the self, personal goals) (Stephens et al., 2012a). In contrast, continuing generation college students (whose parents have received a bachelor's degree) tend to enter college with cultural values aligned with the university's culture of independence (Stephens et al., 2012a). This is important because the experience of cultural mismatch is theorized to predict poorer health during the transition to college, and could potentially even increase future health risks (e.g., by accelerating biological aging). Supporting this, experimental research has shown that cultural mismatch can elevate cortisol levels when students are placed in a broad cultural mismatch situation that primes independent values of the university (vs. priming of interdependent values; Stephens et al., 2012b). Our work builds on this literature by extending the scope of health outcomes studied – specifically, by examining a novel biomarker of health: epigenetic age acceleration. We investigate the association between one particular form of cultural mismatch (home-school cultural value mismatch) and epigenetic age acceleration in a sample of historically marginalized students predominately from low socioeconomic status homes. This sample is theorized to hold strong interdependent values for family (Covarrubias, 2021; Greenfield, 2009; Vasquez-Salgado et al., 2021), and therefore, a greater likelihood of internalizing cultural mismatch between family and academic obligations. Indeed, the interdependent values in the current sample are significantly higher (*M* = 4.4) than that of a higher socioeconomic status sample (*M* = 3.8; Vasquez-Salgado et al., 2021), underscoring the relevance of studying cultural mismatch in this context.

### 1.3 Current study

Based on cultural mismatch theory (Stephens et al., 2012a) and literature (Vasquez-Salgado et al., 2015, 2018, 2021), as well as seminal work linking psychosocial stress to epigenetic aging (Holloway et al., 2023; Palma-Gudiel et al., 2020; Potente et al., 2023), the purpose of the current study was to examine the association between home-school cultural value mismatch and epigenetic aging during the transition to college. We hypothesized that higher levels of reported cultural mismatch would be associated with accelerated epigenetic age.

## 2 Method

### 2.1 Sample

First-year students from historically marginalized backgrounds were recruited for a Transition to College Study during their first semester at a four-year public university in Los Angeles County. Recruitment was completed via direct emails, flyers and social media. One hundred and eleven participants accepted our invitation to join our study, and 94 completed all components (online survey, saliva collection, body-mass-index assessment). A Pilot Project grant from the USC/UCLA Center on Biodemography and Population Health allowed for assay of 66 salivary samples for epigenetic age, 2 of which were subsequently omitted due to technical invalidity (insufficient DNA, and a misalignment between stated biological sex and DNAm-implied biological sex). Thus, the final sample size consisted of 64 participants, with 65.6 % (*n* = 42) identifying as female and 34.4 % (*n* = 22) as male. Fifty-three (82.8 %) identified as Latinx, and 11 (17.2 %) identified as Black, with an average age of 18.0 (*SD* = 0.4). The average parental education and income reported was some high school and $30,000-$39,999 per year, respectively. Finally, most of the sample were first-generation college students (87.5 %; *n* = 56). See Table 1 for a comprehensive list of participants’ characteristics.

**Table 1:** Participant demographics (N = 64) and descriptive statistics for all variables of interest.

Variables	*N*	*M (SD)* or %
Cultural Mismatch 1.1–4.5	64	2.7 (0.8)
Chronological Age 17–19	64	18.0 (0.4)
Epigenetic Age		
GrimAge 18.6–39.4	64	27.2 (4.6)
FitAge 8.7–38.1	64	19.6 (6.4)
Epigenetic Age Acceleration		
GrimAge acceleration .6–21.4	64	9.2 (4.5)
FitAge acceleration −9.3 – 20.1	64	1.6 (6.3)
DunedinPACE .8–1.9	64	1.2 (0.3)
Ethnicity		
Latinx	53	82.8 %
Black	11	17.2 %
Biological Sex		
Male	22	34.4 %
Female	42	65.6 %
BMI 17.6–48.3	64	25.5 (5.5)
First-Generation College Student Status		
First-generation college student	56	87.5 %
Not first-generation college student	8	12.5 %
Mother's Level of Education		
No formal education	1	1.6 %
Some elementary school	6	9.5 %
Elementary school	4	6.3 %
Some junior high school	10	15.9 %
Some high school	4	6.3 %
Graduated from high school	14	22.2 %
Some college or postsecondary education	15	23.8 %
A vocational degree or an associate degree	3	4.8 %
Bachelor's degree	4	6.3 %
Master's degree	2	3.2 %
Missing or don't know	1	
Father's Level of Education		
No formal education	3	5.1 %
Some elementary school	4	6.8 %
Elementary school	3	5.1 %
Some junior high school	6	10.2 %
Junior high school	3	5.1 %
Some high school	10	16.9 %
Graduated from high school	17	28.8 %
Some college or postsecondary education	7	11.9 %
A vocational degree or an associate degree	2	3.4 %
Bachelor's degree	4	6.8 %
Missing or don't know	5	
Parental Income		
< $10,000	5	7.8 %
$10,000 – $19,999	10	15.6 %
$20,000 – $29,999	17	26.6 %
$30,000 – $39,999	5	7.8 %
$40,000 – $49,999	6	9.4 %
$50,000 – $59,999	3	4.7 %
$60,000 – $69,999	6	9.4 %
$70,000 – $79,999	5	7.8 %
$80,000 – $89,999	3	4.7 %
$100,000 – $149,999	2	3.1 %
$150,000 – $199,999	2	3.1 %
Smoking and Alcohol		
Yes	3	4.7 %
No	61	95.3 %

*Note*. There were no missing values in this dataset. One exception is a total of six participants had missing values for either mother (*n* = 1) or father's (*n* = 5) level of education, as they left the field blank or responded with “don't know.” In these instances, the other parent's education level was used to determine first-generation college status.

### 2.2 Procedure

Students interested in joining our study completed an online prescreening consisting of demographic questions and a 10-item health questionnaire to screen for various health conditions (e.g., cardiovascular conditions, respiratory issues, autoimmune conditions, conditions of inflammation, gastrointestinal conditions). Students who were in their first year of study at the university, considered a freshman, identified with a historically marginalized background (e.g., Latinx, Black), and did not have any underlying health conditions or learning disabilities, were invited to participate in the study via email. Those interested in participating received a digital consent form detailing an overview of the study. Once consent was provided, students were directed to complete an online survey. Subsequently, participants took part in a secondary health screening and those that continued to meet criteria met in person with a researcher from our laboratory to review and sign a separate consent form for their engagement in a health activity involving saliva collection, a self-report survey of their health behaviors, and body-mass index using a Tanita scale. Those that agreed deposited saliva into a 1.8 mL cryovial using a saliva collection aid. Participants were given 2 min to fill the vial to the 1.8 mL fill line; if they were unable to do so, they were given an additional 2 min, with a maximum of 4 min to reach the required volume. Prior to the collection of the sample, participants were instructed to rinse their mouths with water to remove any residue, lipstick, or Chapstick that may be present, and then to withhold from eating, drinking, and chewing gum for at least 10 min before sample collection. After the sample was collected, it was immediately placed in a −20 °C freezer. Once data collection was complete, all samples were moved to a −80 °C freezer where they remained until transported for epigenetic analysis at the University of California, Los Angeles (UCLA) Social Genomics Core Laboratory. Salivary DNA was extracted from samples using the manufacture's standard protocol for saliva (Qiagen QIAamp DNA Micro Kit) and 600 ng of total DNA was subject to genome-wide methylation profiling using Illumina Methylation EPIC V2 microarrays, with all assay procedures performed by the UCLA Neuroscience Genomics Core following the manufacturer's standard protocol. The resulting genome-wide DNAm profiles (.IDAT files) were submitted with associated age and sex annotation data to the ClockFoundation.org online scoring system to compute GrimAge, FitAge and DunedinPACE values (described below).

### 2.3 Measures

***Home-School Cultural Value Mismatch***. We utilized a 14-item scale that measures the extent students experience mismatch between one's family and academic obligations. Items were introduced with the statement: “Since you started at [Institution], how often have you had to choose between your academic work and the following things …”. Sample items included: “attending family events,” “doing tasks your family needs done,” and “helping take care of family members (e.g., grandparents, parents, siblings)”. A 5-point Likert scale ranging from 1 = *Never* to 5 = *Very Frequently* was used to gather responses. The Cronbach's alpha for this scale was .85. The items in this cultural mismatch scale were based on the lived experiences of Latinx first-generation college students (Vasquez-Salgado et al., 2015) and broader family obligation literature (Fuligni et al., 1999). The scale was validated with a multi-ethnic sample (for a complete list of the items and psychometric properties, please see Vasquez-Salgado et al., 2021).

***Epigenetic Age Acceleration.*** We employed two second-generation clocks (GrimAge and FitAge) and one third-generation clock (DunedinPACE). Among the second-generation clocks, GrimAge was trained on plasma proteins associated with mortality and disease risk, as well as smoking pack years (Lu et al., 2019), while FitAge was trained on fitness parameters (e.g., mobility, strength, lung function, cardiovascular fitness; McGreevy et al., 2023). In contrast, DunedinPACE was trained using longitudinal changes in multi-organ-system biomarkers (e.g., cardiovascular, metabolic, renal, etc.), resulting in estimates on the rate of decline in system integrity (Belsky et al., 2022). GrimAge, FitAge and DunedinPace are similar in that they have demonstrated ability to predict disease risk and mortality (Belsky et al., 2022; Lu et al., 2019; McCroy et al., 2021; McGreevy et al., 2023). GrimAge acceleration (i.e., GrimAge – chronological age) and FitAge acceleration are on the same metric such that they have an interpretable 0 that denotes when chronological and epigenetic age are the same; positive differences between epigenetic age and chronological age indicate accelerated biological aging while negative differences suggest slower biological aging (Zavala et al., 2024). DunedinPACE is on a slightly different metric such that a 1 represents a 100 % pace of biological aging alignment with chronological aging, and a value of 1.1 implies a 10 % acceleration in pace of biological aging, and a .9 implies a pace of biological aging that is 10 % slower (Gehle et al., 2023).

***Covariates*.** All analyses controlled for chronological age, ethnicity (Black coded as “0” and Latinx coded as “1”), biological sex (female coded as “0” and male as “1”), body-mass-index (BMI), socioeconomic status (i.e., first-generation college status; parental income), and smoking and alcohol use (no coded as “0” and yes coded as “1”). We controlled for these variables because prior research has indicated their strong influence on epigenetic age acceleration (Crimmins et al., 2021; Harris et al., 2024; Kankaanpää et al., 2021; Schmitz et al., 2022). First-generation college status was calculated by reviewing mother and fathers' level of education for each student. Those with at least one parent that had a bachelor's degree were coded as “0” = not a first-generation college student and those with parents that did not receive a bachelor's degree were coded as “1” = first-generation college student (Stephens et al., 2012b, Stephens et al., 2012aa). BMI was assessed using a Tanita scale. Height measurements were taken in triplicate with a SECA 213 stadiometer, entered onto the scale, and thereafter, the participant stood evenly on the Tanita platform for their BMI to be gathered.

Demographic variables (chronological age, ethnicity, biological sex, socioeconomic status) were gathered via a self-report survey at entry into the study, and smoking and alcohol use (“Have you smoked or consumed alcohol within the last 24 h?”) was gathered via a self-report survey towards the conclusion of their participation in the study. Though we did not assess for typical smoking and alcohol consumption behaviors, most smokers and alcohol consumers are habitual/chronic, so it was expected that their responses would be consistent over time (Albery et al., 2015; Birge et al., 2018).

### 2.4 Analysis plan

Three separate hierarchical linear regressions were conducted to examine the unique contribution of home-school cultural value mismatch to each of the epigenetic age acceleration measures (GrimAge, FitAge, and DunedinPACE). A two-step approach was employed to control for potential covariates in Step 1 (chronological age, ethnicity, biological sex, BMI, first-generation college status, parent income, and smoking and alcohol consumption) and assess whether home-school cultural value mismatch accounted for additional, unique variance in epigenetic age acceleration in Step 2. The significance of home-school cultural value mismatch was evaluated through the regression coefficient and the change in R^2^ (ΔR^2^), indicating its unique effect and improvement in model fit, respectively (Tabachnick and Fidell, 2019). To facilitate interpretation, cultural mismatch, chronological age, BMI, and parent income were standardized (*M* = 0, *SD* = 1). Analyses were conducted using IBM SPSS Version 29.

## 3 Results

Our key variables of investigation (cultural mismatch and epigenetic age acceleration measured by GrimAge, FitAge, DunedinPACE) were examined for normality, and all were within an acceptable range (skewness and kurtosis between −2 and 2). All epigenetic aging measures indicated an average age acceleration in this sample. For GrimAge, the average epigenetic age was 27.2 (*SD* = 4.6), and average epigenetic age acceleration was 9.2 (*SD* = 4.5). For FitAge, the average epigenetic age was 19.6 (*SD* = 6.4), and average epigenetic age acceleration was 1.6 (*SD* = 6.3). Together, this implies that on average, the sample was biologically 9 years older than their chronological age of 18 vis a vis GrimAge (a disease risk and mortality-driven measure), and 1.6 years older than their chronological age of 18 vis a vis FitAge (a fitness-driven measure). DunedinPACE averaged 1.2 (*SD* = 0.3), implying that participants in this sample were aging 20 % faster than the average rate of the population. See Table 1 for descriptives of all variables of interest.

Preliminary analysis revealed marginally significant positive zero-order correlations between home-school cultural value mismatch and two of the three epigenetic age acceleration measures: GrimAge, *r*(64) = .23, *p* = .067; FitAge *r*(64) = .23, *p* = .065; DunedinPACE *r*(64) = .14, *p* = .279. Among the covariates, chronological age was marginally associated with all epigenetic age acceleration measures: GrimAge, *r*(64) = .23, *p* = .065; FitAge *r*(64) = .22, *p* = .086; DunedinPACE *r*(64) = .22, *p* = .083. In addition, our measure of smoking and alcohol consumption exhibited a moderate positive association with all three epigenetic acceleration measures: GrimAge *r*(64) = .30, *p* = .017; FitAge *r*(64) = .29, *p* = .019; DunedinPACE *r*(64) = .31, *p* = .012. Being male was significantly associated with greater FitAge acceleration, *r*(64) = .25, *p* = .047, but there were no differences in acceleration for GrimAge, *r*(64) = .20, *p* = .117, or DunedinPACE, *r*(64) = .07, *p* = .566. Being a first-generation college student was marginally associated with slower FitAge acceleration, *r*(64) = -.24, *p* = .057, but no differences in acceleration were observed for Grim Age, *r*(64) = -.16, *p* = .200, or DunedinPACE, *r*(64) = -.20, *p* = .117. Other covariates, including ethnicity, BMI, and parent income, were not significantly associated with the epigenetic age acceleration measures (*p*_s_ = .135 - .771). Nonetheless, we controlled for all covariates in our analyses as these are typical controls in epigenetic age research.

**Fig. 1:**
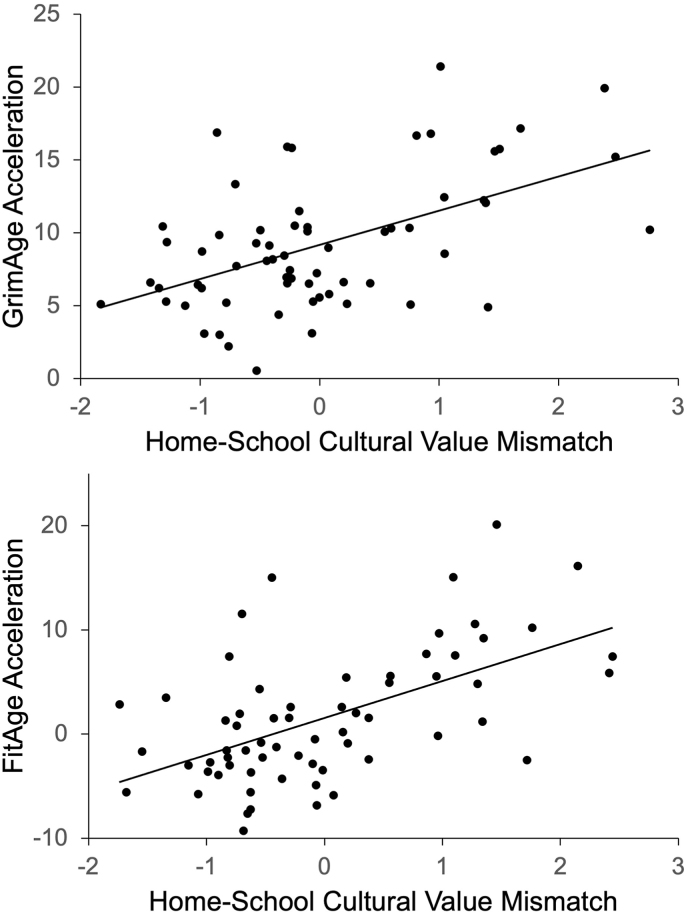
Home-School Cultural Value Mismatch as a Predictor of Epigenetic Age Acceleration *Note*. Home-School Cultural Value Mismatch scores were standardized. Age, BMI, and parent income were standardized and included as covariates. Ethnicity, biological sex, first-generation college status, and smoking and alcohol use were additional covariates. Ethnicity was coded as 0 = Black and 1 = Latinx. Biological sex was coded as 0 = Female and 1 = Male. First-generation college status was coded as 0 = No and 1 = Yes. Smoking and alcohol use was coded as 0 = No and 1 = Yes.

To test the hypothesis that higher levels of reported home-school cultural value mismatch would be associated with accelerated epigenetic aging, hierarchical linear regressions were modeled with control variables (chronological age, ethnicity, biological sex, BMI, first-generation college status, parent income, smoking and alcohol consumption) in Step 1 and cultural mismatch in Step 2. As expected, higher levels of cultural mismatch predicted accelerated epigenetic aging (GrimAge, FitAge) among historically marginalized students during the transition to college (Table 2). For every 1-SD increase in cultural mismatch experienced, epigenetic age acceleration increased by approximately 1.3 and 2.0 years, respectively (Fig. 1). In addition, the total variance explained in GrimAge and FitAge was 27 % and 32 %, respectively, with 8 to 9 % of this variance reflecting the unique, significant contribution of home-school cultural value mismatch and improvement of model fit. However, DunedinPACE did not vary as a function of home-school cultural value mismatch (Table 2).

**Table 2:** Hierarchical linear regression models assessing home-school cultural value mismatch as a predictor of epigenetic age acceleration.

	B	SE	β	R^2^	ΔR^2^
**GrimAge Acceleration**					
Step 1				.19+	
Age	.67	.58	.15		
Ethnicity (Latinx)	.04	1.44	.00		
Biological sex (male)	1.16	1.21	.12		
BMI	.36	.57	.08		
First-gen. college status	−2.59	1.78	−.19		
Parent income	−.40	.58	−.09		
Smoking and alcohol	6.33∗	2.61	.30		
Step 2				.27∗	.08∗
Cultural mismatch	1.32∗	.55	.29		
**FitAge Acceleration**					
Step 1				.23∗	
Age	.86	.79	.14		
Ethnicity (Latinx)	.64	1.98	.04		
Biological sex (male)	2.21	1.66	.17		
BMI	.42	.79	.07		
First-gen. college status	−4.29+	2.42	−.23		
Parent income	.23	.80	.04		
Smoking and alcohol	8.68∗	3.59	.29		
Step 2				.32∗∗	.09∗∗
Cultural mismatch	2.01∗	.75	.32		
**DunedinPACE**					
Step 1				.25∗	
Age	.04	.03	.15		
Ethnicity (Latinx)	.08	.08	.12		
Biological sex (male)	−.01	.07	−.02		
BMI	.03	.03	.13		
First-gen. college status	−.22∗	.10	−.29		
Parent income	−.05	.03	−.18		
Smoking and alcohol	.36∗	.14	.31		
Step 2				.28∗	.03
Cultural mismatch	.04	.03	.17		

*Note*. Home-School Cultural Value Mismatch scores were standardized. Age, BMI, and parent income were standardized and included as covariates. Ethnicity, biological sex, first-generation college status, and smoking and alcohol use were additional covariates. Ethnicity was coded as 0 = Black and 1 = Latinx. Biological sex was coded as 0 = Female and 1 = Male. First-generation college status was coded as 0 = No and 1 = Yes. Smoking and alcohol use was coded as 0 = No and 1 = Yes. ∗∗*p* < .01, ∗*p* < .05, +*p* < .10.

## 4 Discussion

We investigated associations between home-school cultural value mismatch – a mismatch between family and academic obligations – and three epigenetic-clock measures of biological aging in a sample of historically marginalized students enrolled in their first semester at a four-year university. Results revealed that higher levels of home-school cultural value mismatch were associated with *accelerated* *epigenetic aging,* as indicated by two second-generation epigenetic clocks, GrimAge and FitAge.

These significant associations emerged even after controlling for covariates, including age, ethnicity, sex, adiposity, smoking and alcohol use, and parental socioeconomic status. These results align with previous qualitative, survey and experimental work documenting a link between home-school cultural mismatch and psychosocial stress (Valle and Covarrubias, 2024; Vasquez-Salgado et al., 2015, 2018, 2021). However, these findings are novel in suggesting that young, historically marginalized students from low socioeconomic households face a cultural barrier during their transition to college that may contribute to accelerated biological aging - potentially elevating future disease risk and mortality (Duan et al., 2022). Our findings extend existing empirical literature linking psychosocial stress and accelerated epigenetic age (Klopack et al., 2022; Palma-Gudiel et al., 2020; Potente et al., 2023; Markon et al., 2024) by identifying sociocultural determinants of biological aging in young, emerging adults.

On average, our 18-year-old sample of historically marginalized students were biologically 9-years older than their chronological age vis a vis GrimAge (a disease risk and mortality-driven measure) and 1.6-years older vis a vis FitAge (a fitness-driven measure), underscoring the importance of understanding the biological pathways involved in socioeconomic and cultural health disparities as research seeks to promote health equity across the entire population. Results also indicated that students in this sample were aging 20 % faster than the population vis a vis DunedinPACE, a third-generation clock representing the pace of aging (although this association did not reach statistical significance). The heterogeneity of our findings aligns with the notion that different epigenetic clocks capture distinct aspects of biological aging (Belsky et al., 2018).

Our findings may have important implications for future research and intervention. Future research in larger and more nationally-representative samples is needed to validate the present observations linking cultural mismatch and accelerated epigenetic age, and to identify the mediating processes that connect them together. In terms of intervention, there is a need for higher-education stakeholders (faculty, programs, administrative offices) to receive cross-cultural sensitivity training on cultural mismatch as well as strategies that can be used to support students facing these barriers (Vargas et al., 2024). This can pave the way for improved policies, interactions and programmatic efforts to harmonize between home and academic contexts and reduce experiences of mismatch. For example, this can involve something as simple as a being more understanding and flexible with students experiencing a cultural mismatch between family and academic obligations. It can also involve a university-wide campaign that motivates students to share their educational experiences with their parents or an “active” open house that enables parents to attend courses and activities of first-time freshmen for one day. In doing so, the home and school contexts (equally important facets of students’ development) can work towards becoming more interconnected, flexible and understanding of one another. Historically marginalized students are a growing population that make up the next generation of professionals and workforce, and the health implications of home-school cultural mismatch during the transition to college is a key concern in the context of population health equity.

It is important to note limitations of the current study. First, the sample was relatively small, and the findings are limited to the types of students recruited: healthy, historically marginalized first-time freshmen at a public, four-year university in Los Angeles County. Therefore, generalizability remains to be determined with more diverse samples. Second, it is important to acknowledge the limitations of using saliva as a tool for assessing epigenetic age acceleration. Though saliva is convenient and non-invasive, it may not capture the full diversity of cell types within tissues, such as blood, potentially influencing the accuracy of age estimations (Apsley et al., 2025). Lastly, results come from a cross-sectional/correlational analysis, and thus cannot definitively indicate that home-school cultural mismatch causes accelerated epigenetic aging (reverse causation may be possible, as would confounding effects with other factors that influence both). Future research using experimental interventions with collection of salivary and blood samples will be required to definitively identify causal effects and rigorously map their psychobiological mechanism.

## 5 Conclusion

Our findings identify a novel connection between home-school cultural value mismatch and accelerated epigenetic aging among historically marginalized college students. Because accelerated epigenetic age predicts future disease risk and mortality, more research is needed to quantify the health impacts of cultural mismatch and develop and put into action new interventions that foster the harmonization between home and academic cultures.

## CRediT authorship contribution statement

**Yolanda Vasquez-Salgado:** Writing – review & editing, Writing – original draft, Visualization, Validation, Supervision, Resources, Project administration, Methodology, Investigation, Funding acquisition, Formal analysis, Data curation, Conceptualization. **Gabrielle Halim:** Writing – review & editing, Writing – original draft, Visualization. **Angel E. Morales:** Writing – review & editing, Writing – original draft. **Katelan Galvan:** Writing – review & editing, Writing – original draft. **Teresa Seeman:** Writing – review & editing, Writing – original draft, Formal analysis, Conceptualization. **Steve Cole:** Writing – review & editing, Writing – original draft, Software, Methodology, Formal analysis, Data curation, Conceptualization.

## Ethics approval

This study aligns with and was approved by the Institution's IRB. A document of informed consent was provided to all participants of this study.

## Funding

Support of this manuscript has been provided by the following: the 10.13039/100000057National Institute of General Medical Sciences of the 10.13039/100000002National Institutes of Health under Award Numbers RL5GM118975, TL4GM118977, UL1GM118976, GM136450, and R16GM146693. Instrumental support has also been provided by the USC/UCLA Center on Biodemography and Population Health through the 10.13039/100000049National Institute on Aging (NIA) Grant Number P30AG017265. The content is solely the responsibility of the authors and does not necessarily represent the official views of the National Institutes of Health.

## Declaration of competing interest

There are no conflict of interest among all authors.

## Data Availability

Data will be made available on request.
